# Role of ROS in Aβ42 Mediated Activation of Cerebral Endothelial Cells

**DOI:** 10.5195/cajgh.2014.179

**Published:** 2014-12-12

**Authors:** Andrey Tsoy, Bauyrzhan Umbayev, Tamara Shalakhmetova, Sholpan Askarova

**Affiliations:** 1 Center for Life Science, Nazarbayev University, Astana, Kazakhstan; 2 Department of Biodiversity and Bioresources, Al-Farabi Kazakh National University, Almaty, Kazakhstan

**Keywords:** Alzheimer’s disease, cerebral endothelial cells, ROS, Aβ4, P-selectin

## Abstract

**Introduction:**

There is substantial evidence that the deposition of aggregated amyloid-beta peptide (Aβ) in brain parenchyma and brain vessels is the main cause of neuronal dysfunction and death in Alzheimer’s disease (AD). Aβ exhibits multiple cytotoxic effects on neurons and glial cells and causes dysfunction of the blood brain barrier (BBB). In AD brains, an increased deposition of Aβ in the cerebral vasculature has been found to be correlated with increased transmigration of blood-borne inflammatory cells and neurovascular inflammation. However, regulatory mediators of these processes remain to be elucidated. In this study, we examined the role of ROS in actin polymerization and expression of adhesion molecules (P-selectin) on the surface of the cerebral endothelial cells (CECs) that are activated by Aβ42.

**Materials and methods:**

Mouse BEnd3 line (ATCC) was used in this research. BEnd3 cells respond to Aβ treatment similarly to human primary CECs and are a common model to investigate CECs’ function. We used immortalized bEnd3 cells as the following: controls; cells incubated with Aβ42 for 10, 30, and 60 minutes; cells incubated with 30 mM of antioxidant N-acetylcysteine (NAC) for 1 hr; and, cells pre-treated with NAC followed by Aβ42 exposure. We measured DHE fluorescence to investigate intracellular ROS production. Immunofluorescent microscopy of anti-P-selectin and oregon green phalloidin was used to quantify the surface P-selectin expression and actin polymerization, and Western blot analysis was used to analyze total P-selectin expression.

**Results:**

The results of this study have demonstrated a significant time-dependent ROS accumulation after 10 minutes, 30 minutes, and 60 minutes of Aβ42 treatment, while Aβ42 stimulated ROS production in CECs was attenuated by pre-treatment with the NAC antioxidant. We also found that Aβ42 increased P-selectin fluorescence at the surface of bEnd3 cells in a time dependent manner in parallel to ROS elevation. However, total expression levels of P-selectin were not changed following exposure to Aβ42. Pretreatment with NAC attenuated Aβ42 induced P-selectin localization, while NAC alone did not significantly affect P selectin localization. As a positive control, H_2_O_2_ also increased P-selectin expression on the cell surface, which peaked after 30 minutes of H_2_O_2_ treatment. Exposure of CECs with Aβ42 promoted actin polymerization, which peaked after 10 minutes of Aβ42 treatment, while no significant increase of F-actin intensity was observed when cells were pre-treated with NAC. H_2_O_2_ was able to mimic Aβ42 induced oxidative stress, causing increased actin polymerization with similar timing.

**Conclusions:**

The results of our study have indicated that Aβ42 induced accumulation of P-selectin on the surface of bEnd3 cells and promoted actin polymerization, and all these events were correlated with ROS generation. The rapid post-translational cell signaling response mediated by ROS may well represent an important physiological trigger of the microvascular inflammatory responses in AD and requires further investigations.

